# Non-suicidal self-injury (Nssi) in adolescent inpatients: assessing personality features and attitude toward death

**DOI:** 10.1186/1753-2000-6-12

**Published:** 2012-03-30

**Authors:** Mauro Ferrara, Arianna Terrinoni, Riccardo Williams

**Affiliations:** 1 Department of Pediatrics and Child and Adolescent Neurology and Psychiatry, "Sapienza" University of Rome, Via dei Sabelli 108, 00185 Rome, Italy; 2 Department of Dynamic and Clinical Psychology, "Sapienza" University of Rome, Via degli Apuli 1, 00185 Rome, Italy

**Keywords:** Non-suicidal self-injury (NSSI), Suicide, Attempted, Adolescent psychiatry, Inpatients, Borderline personality disorder

## Abstract

**Background:**

Non-suicidal self-injury (NSSI) is a common concern among hospitalized adolescents, and can have significant implications for short and long-term prognosis. Little research has been devoted on how personality features in severely ill adolescents interact with NSSI and "attitude toward life and death" as a dimension of suicidality. Developing more specific assessment methodologies for adolescents who engage in self-harm without suicidal intent is relevant given the recent proposal of a non-suicidal self-injury (NSSI) disorder and may be useful in predicting risk in psychiatrically impaired subjects.

**Methods:**

Consecutively hospitalized adolescents in a psychiatric unit (N = 52; 71% females; age 12-19 years), reporting at least one recent episode of self-harm according to the *Deliberate Self-harm Inventory*, were administered the *Structured Clinical Interview for DSM Mental Disorders and Personality Disorders (SCID I and II)*, the *Children's Depression Inventory *and the *Multi-Attitude Suicide Tendency Scale (MAST)*.

**Results:**

Mean age onset of NSSI in the sample was 12.3 years. All patients showed "repetitive" NSSI (high frequency of self-harm), covering different modalities. Results revealed that 63.5% of adolescents met criteria for Borderline Personality Disorder (BPD) and that the rest of the sample also met criteria for personality disorders with dysregulated traits. History of suicide attempts was present in 46.1% of cases. Elevated depressive traits were found in 53.8%. Results show a statistically significant negative correlation between the score on the "Attraction to Life" subscale of the MAST and the *frequency *and *diversification *of self-harming behaviors.

**Conclusions:**

Most adolescent inpatients with NSSI met criteria for emotionally dysregulated personality disorders, and showed a reduced "attraction to life" disposition and significant depressive symptoms. This peculiar psychopathological configuration must be addressed in the treatment of adolescent inpatients engaging in NSSI and taken into account for the prevention of suicidal behavior in self-injuring adolescents who do not exhibit an explicit intent to die.

## Background

Research on adolescent development has devoted efforts to the understanding of the roots of potentially self damaging behaviors, including suicide, eating disorders, substance abuse, sexual promiscuity, risk-taking, violence and aggression, delinquency and, more recently, self-harm or non-suicidal self-injurious behavior (NSSI). Literature data have shown that adolescence is a critical period for the onset of self-harm [1]. The prevalence of NSSI among adolescents in community based studies range between 13% and 28% [2-4]; in general, literature suggests a seemingly increasing prevalence of such behaviors in the teenager population [5,6].

Not surprisingly, higher rates of self-harm are apparent in individuals receiving mental health treatment: NSSI occurs in about 20% of adult psychiatric patients [7] and in 40-80% of adolescent psychiatric patients [8-10].

NSSI has also been described as one of the most diffuse and challenging clinical phenomena reported in adolescent inpatient samples [11,12]. A recent review study of discharge diagnoses indicated a threefold increase in NSSI among hospitalized adolescents from 1990 to 2000 [13].

Much debate concerns the psychopathological meaning of NSSI, due to its uncertain boundaries and heterogeneous manifestations. Overall, literature on psychopathological characteristics of NSSI in adolescence has considered separately two distinct controversial aspects: the relationship between NSSI and personality disorders, and the relationship between NSSI and the depressive-suicidal dimension. Moreover, adolescents with severe personality disorders are often assessed and treated in different clinical settings and with different approaches than those with mood disorders and suicidality.

Empirical findings show that the psychopathological dimension more consistently related to NSSI concerns personality functioning. In particular, a close link has been evidenced between NSSI and Borderline Personality Disorder (BPD) [14-17]. The majority of studies concern adult population. Indeed, epidemiological data show that 80% of adult BPD patients have exhibited at least one episode of self-harm [18]. In DSM-IV [19], self-harm has been represented under criterion 5 of BPD: keeping in mind the controversies about diagnosing personality disorders prior to adulthood and the fact that considering self-harm pathognomonic of BPD could lead to inappropriate management, the "DSM-5 Childhood and Adolescent Work Group" is now recommending the inclusion of the new diagnosis: *Non-suicidal Self-Injury (NSSI)*. The proposed new diagnosis of NSSI applies to individuals engaging in intentional self-inflicted damage on 5 or more days in the last year, without suicidal intent and presenting a significant distress or impairment. The inclusion of the new diagnosis may reflect the clear cut relation between NSSI and childhood/adolescence, reduce the automatic assumption that an adolescent who engages in NSSI may have BPD and hopefully promote research to further clinical guidelines for treatment [20,21]. Since NSSI is a distinct aspect from BPD, it is important to fully articulate the relationship between NSSI and personality functioning in adolescence. The analysis of personality features of adolescent inpatient and outpatient populations exhibiting NSSI have so far confirmed the typical adult association between NSSI and BPD [21-23].

Substantial research attention has been given to the presence of specific forms of psychopathology associated with NSSI among hospitalized adolescent: it has been suggested [22] that most adolescent inpatients engaging in NSSI meet criteria for a DSM-IV Axis I diagnosis, with elevated rates of Major Depressive Disorder (MDD) (42%), Post-Traumatic Stress Disorder (PTSD) (24%), Substance Use Disorder (SUD) (60%). In a retrospective chart review using medical records, Jacobson et al. [23] found 67% of MDD in the total sample examined (NSSI and NSSI "plus suicide attempts" outpatients); Muehlenkamp et al. [24], using a similar methodology, examined how BPD symptoms relate to suicide attempts or NSSI within a population of adolescent outpatients, finding two BPD features ("confusion about self" and "unstable interpersonal relationships") as distinct predictors of "NSSI" and "NSSI + Suicide" group status, but not a strong variation in the impact of the single features on the different subgroups.

Although the inclusion of the new diagnosis makes clear the intention to consider NSSI and suicide attempts as distinct phenomena, several important questions are yet to be explained. First, NSSI and suicide attempts could co-occur with different modalities in different clinical populations: a sizable portion of self-injurers (50% of outpatients; 70% of inpatients) reports having attempted suicide at least once [5-22]. Some epidemiological and research data evidence that many suicides are not preceded by NSSI. In general population samples NSSI seems to have less severe consequences than attempted suicide and a different risk trajectory [5,6], but in adolescent inpatients who have attempted suicide a history of NSSI before the index episode is more likely than in those who have only suicidal ideation [25]. Data from ADAPT study show that in depressed adolescents receiving treatment over a 6 months follow-up, NSSI at baseline is an independent predictor of suicide attempt, even stronger than a history of suicide attempt itself [26]; Asarnow and colleagues identified similar findings in adolescents with treatment- resistant depression [27].

Second, although prior research has focused on the identifications of possible psychopathological links between NSSI, depression and suicidal ideation this crucial question remains unclear. Depressive symptoms seem to distinguish "NSSI-only" patients from NSSI patients who attempt suicide [23], thus implying a role for depressive conditions in the escalation from NSSI to suicidal behaviors. Moreover, self-harm has been found to be associated with depressive ideation, including feeling repulsed by life, having greater amounts of apathy, self-blame, and fewer connections to family members [28]. By definition, explicit suicidal ideation would not pertain NSSI subjects who, nonetheless, may end up in attempting suicide. Therefore, the individuation of instruments aimed in helping clinicians identify a suicide risk when there is not an explicit suicidal ideation seems a necessary step. Ideational factors related to feelings toward life and death has proved a useful construct in discriminating suicidal adolescents, non-suicidal adolescents and a psychiatric control group [29].

Given these considerations in this study we pursue two main objectives:

1) To describe the characteristics of NSSI and related psychopathology/personality functioning in a sample of NSSI adolescent inpatients;

2) To investigate whether characteristics such as depression symptoms and attitude toward life and death discriminate between NSSI subjects who have attempted suicide (NSSI - SA) and NSSI subjects who have not attempted suicide (NSSI only).

## Methods

The participants for this study were consecutively admitted adolescents to a psychiatric inpatient unit ("Sapienza", University of Rome) that specializes in treating severe behavioral problems and psychotic episodes. A total of 114 adolescents were admitted in the study period (September 2009-September 2010), the majority of whom presented severe disruptive and/or personality disorders. All youths who presented NSSI over a 12-month period preceding admission were included. All adolescents with an attempted suicide as admission diagnosis were triaged to a separate unit and not included. Patients diagnosed with intellectual disabilities, pervasive developmental disorders, schizophrenia spectrum disorders or associated neurological conditions were excluded, given the relevance of such conditions for stereotypic self-injuries. The selected sample consisted of 52 adolescents (15 males and 37 females (age 12-19: mean = 15.50, SD = 1.72; age females: mean = 15.50, SD = 1.62; age males: mean = 15.2, SD = 2.03).

The great majority of the sample was made of ethnically white Italian. Of the remaining participants, 3 were Asian and 1 was unidentified. The distribution is reflective of the ethnic composition of the surrounding community.

As a standard assessment procedure, all subjects were interviewed and screened for DSM Axis I and Axis II diagnoses via the *Structured Clinical Interview for DSM Mental Disorders and Personality Disorders (SCID I and II) *[30]; the participants were administered the study measures, including the *Deliberate Self-harm Inventory (DSHI)*, the *Children's Depression Inventory (CDI) *and the *Multi-Attitude Suicide Tendency Scale (MAST)*. Youths and their parents signed informed consent for the evaluation. The study did not require any formal authorization, since the hospital's institutional review board did not consider the procedure as invasive.

The following instruments were administered sequentially for each patient, in a series of three sessions requiring 50 min each.

### Instruments

The Deliberate Self-harm Inventory (DSHI) [31] was designed to measure non-suicidal deliberate self-harm; it is a self-report questionnaire that measures frequency, age of onset, duration, date of last occurrence, and severity of 17 types of self-harming behavior. The subject is required to report the relative frequency of each type of behavior on a 5-level scale: never-seldom-sometimes-often-always. The DSHI has adequate internal consistency (α = 0.82), temporal reliability (r = 0.92), and support for validity. For descriptive purposes we used the indexes of *frequency *= number of episodes-per-month (seldom = episodic self-harm; sometimes to always = repetitive self-harm), *types *of self-harming behaviors (e.g., self-cutting, self-burning, etc.), *diversification *= occurrence of multiple types of self-harming behaviors measured on a three-level scale (0-1 types: minimum diversification; 2-4: moderate diversification; and 5-11: high diversification).

Children's Depression Inventory (CDI). This scale was developed by Kovacs [32] in order to assess the level of childhood depressive symptoms. It contains 27 items, each of which consists of 3 statements. For each item, the participant is asked to select the statement that best describes his/her feelings during the past 2 weeks. Scores range from 0 to 54. Higher scores indicate more severe depression.

The Multi-Attitude Suicide Tendency (MAST) [29] scale is a 30 item self-report measure of adolescent attitude toward life and death. The four types of conflicting attitudes identified are Attraction to Life (AL, 7 items), Repulsion by Life (RL, 7 items), Attraction to Death (AD, 7 items) and Repulsion by Death (RD, 9 items). The MAST was developed and initially validated in 2 studies involving high school and outpatient adolescents [28]. Another study [29] examined the construct validity and psychometric properties of this instrument in adolescent psychiatric inpatients and suggested that at least three of the MAST subscales may contribute to the assessment of suicidal behavior in adolescent psychiatric inpatients. Aspects concerning robustness and reliability of the Scale are included in Orbach [28].

### Statistical methods

The ANOVA test, Binomial test and Mann Whitney U-test, correlations and linear regression methods were used when appropriate. For descriptive purposes, the results in univariate analyses are reported as significant at ***p ***< 0.05; SPSS software version 15.0 (SPSS Inc., Chicago, IL, USA) was used.

## Results

No missing data were found for any of the participants originally recruited for the study. For all patients included in the study, the NSSI was classified as "repetitive", representing at least 5 lifetime self-harming behaviors on the DSHI. The average frequency of NSSI in the last three months is 2.9 (Table 1) (classified as "often" according to DSHI) The average number of self-injuries types was 5. The DSHI evidenced that 60.5% of the subjects had "high diversification" self-harm (Table 2) "*Cutting" *was the prevalent modality (80.8%), followed by *"Severe scratching" *(57.7%), *"Interference with wound healing" *(50.0%) *and ***"***Sticking pins, needles, staples into skin" *(42.3%). The average age at the onset of NSSI was 12.3 years. Mann-Whitney U test evidenced no statistically significant differences *(p = .689) *for frequency and number of types of NSSI between NSSI subjects who had attempted suicide (NSSI-SA) and NSSI subjects who had not attempted suicide (NSSI only).

**Table 1:** Average number of episodes/ month (e/m) in the last 3 months

Frequency		
**e/m**	**N**	**%**

3	4	5.6%

3 ≥ 6	10	19.2%

6 or more	38	73.2%

Total	52	100%

**Table 2:** Occurrence of multiple types of self-harming behaviors

Diversification		
**Level**	**N**	**%**

0-1 = Minimum	4	8%

2-4 = Moderate	21	41.5%

5-11 = High	27	60.5%

The sample mostly consisted of female subjects (F 71.2%; M 28.8%,). There were statistically significant differences indicating the association between female gender and NSSI (binomial test, *p < 0.05*).

### Psychopathology and personality

Five subjects were diagnosed with an eating disorder (9.6%), 6 (11.5%) subjects with a substance abuse disorder and 10 (19.2%) subjects received a diagnosis for an anxiety disorder (Table 3).

**Table 3:** Sample's characteristics

	NSSI	Male	Female	Total
Country of origin	Italy	12(23.1%)	35(67.3%)	47(90.4%)

	Other countries	3(5.8%)	2(3.8%)	5(9.6%)

Diagnosis Axis II	BPD	5(9.6%)	28(53.9%)	33(63.5%)

	Other Diagnosis (Cluster B)	10(19.2%)	9(17.3%)	19(36.5%)

Diagnosis Axis I	Eating Disorder	0(0.0%)	5(9.6%)	5(9.6%)

	Substance Use Disorder	6(11.3%)	15(29.1%)	21(40.4%)

	Anxiety Disorder	7(13.5%)	3(5.7%)	10(19.2%)

Violence	Physical	5(9.6%)	12(22.8%)	17(32.7%)

	Sexual abuse	2(3.8%)	7(13.5%)	9(17.3%)

The sample shows a strong representation of BPD diagnosis with 63.5% of subjects meeting criteria for this personality disorder. A significant portion of the subjects were otherwise diagnosed with a Cluster B personality disorder: Histrionic personality disorder (*N = 7; 13.5%)*, Narcissistic personality disorder (*N = 3; 5.7*%); 9 subjects were diagnosed with Passive-aggressive personality disorder (*N = 9; 17.3%) *belonging to Cluster C personality disorders. The impact of BPD diagnosis on NSSI was calculated by an ANOVA model having BPD vs other Axis II diagnoses as group predictor and both frequency and number of types derived from DSHI as dependent variables. No statistically significant effects were shown at the ANOVA either for frequency (mean for borderline group = 2.79; mean for non-borderline group = 2.63; SD = .87; F = .338; *p *= .536) and number of types of NSSI (mean for borderline group = 5.27; mean for non-borderline group = 4.22; SD = 2.77; F = 1.695; *p *= .20).

In order to evidence the possible relationship between the severity of depressive symptoms and NSSI, we performed several analyses. First of all, it should be highlighted that half of the patients of the sample (N = 28; 53.8%) had scores that were above the clinical threshold for depressive symptoms on the CDI (clinical cut-off > 19). Second, patients exceeding the cut-off for depressive symptoms at CDI were also significantly more likely to exhibit a greater number of types of NSSI than patients scoring in the non-depressive range of CDI (Mann-Whitney, *p *< 0.05). Finally, it was possible to evidence (see Table 4) a significant positive correlation between CDI scores and number of types of NSSI (r = 397; *p *< .01), while an only non significant positive correlation was found between CDI scores and frequency of NSSI (r = . 206; *p *= .148).

**Table 4:** Statistical Analysis

	Variables	AL	RL	AD	RD
**CDI**	*Pearson*	-.65*	.289*	.360*	**.158**

	*p-value*	**<.01**	**<.01**	**<.01**	**n.s**.

**Types**	*Pearson*	-.293*	**-.074**	**.097**	**-.219**

	*p-value*	**.037**	**.606**	**.498**	**.123**

**Frequency**	*Pearson*	-.287*	**-.027**	**.084**	**-.035**

	*p-value*	*.039	**.850**	**.555**	**.807**

(* = significant value); (AL: Attraction to Life, RL: Repulsion by Life, AD: Attraction to Death and RD: Repulsion by Death)

### Attraction to life and death

Several analyses were also conducted to verify the relationship between attitude toward life and death, NSSI and suicide attempts. Results (see Table 4) show a statistically significant negative correlation between the AL ("Attraction to Life " subscale) scores and the *frequency *and number of types of self-harming behaviors (frequency × AL: *r = -.287, p < .05*; types × AL: r *= -.293, p < .05*). The other subscales of the MAST (Attraction to Death; Repulsion by Life and Repulsion by Death) do not evidence any statistically significant correlations with frequency and number of types of self-harming behaviors.

It has to be noted that the MAST subscales scores and CDI scores show significant associations (CDI × AL: *r = -. 65*, *p < .01*; CDI × RL: *r = .289*, *p < .01*; CDI × AD: *r = .360, p < .01*; CDI × RD: *r = .158, N.S*.) (Table 4). In order to verify whether the significant inverse association between AL scores and frequency and number of types of NSSI was mediated by CDI scores, we performed a linear regression model using frequency and number of types of NSSI as dependent variable and CDI and MAST-AL scores as predictors. The results evidence that the inverse association between AL scores and frequency of NSSI remains significant even when one controls for CDI scores (r = -.287; *p *< .05) and that the variance explained by the interaction between CDI scores and attraction to life scores is not significant (adjusted R2 = .05; F = 2,18; *p *= .124).

A further step of the analyses was to test whether NSSI subjects' CDI scores and scores for all four subscales of the MAST were discriminated by the fact of having or not having attempted suicide. The application of the Mann-Whitney U revealed that belonging to one of the two groups (*NSSI-SA *and *NSSI only*) did not produce any significant difference at both the MAST subscales scores (MAST-AL: NSSI-SA = .2.88, NSSI only = 3,03, SD = .87, z = -.927, *p *= .354; MAST-RL: NSSI-SA = 3.38, NSSI only = 3.04, S.D. = .89, z = -.927, p = .354; MAST-AD: NSSI-SA = 3.26, NSSI only = 2.95, SD = 1.04, z = .641, p = .521; MAST-RD: NSSI-SA = 2.41, NSSI only = 2.16, SD = 1.06, z = .592, *p *= .554) and the CDI scores (NSSI-SA = 20.94, NSSI only = 20.78, SD = 11.42, z = .263; *p *= .793).

## Discussion

This study had two objectives. The first was to describe the characteristics of self-harm and related psychopathology/personality functioning in a sample of NSSI adolescent inpatients. The second objective was to investigate whether depressive symptoms and attitude toward life and death discriminate between NSSI subjects who have attempted suicide and NSSI subjects who have not attempted suicide.

Patients in this sample showed multiple types of self-harming behaviors and displayed a repetitive mode of self-harming. In general, indexes of frequency and diversification should not be considered as indicators of severity, but at the same time they are related to severity of comorbid psychopathology [23] with a mechanism that remains unclear.

The average onset of NSSI in our sample was 12.3 years, indicating a relatively long history of behaviors in the study group. Similar age of onset of NSSI have been found in the general population [2]. However, in this sample young adolescents also manifested frequent and highly diversified self-harm conducts, possibly indicating that adolescents with high rates of psychopathology begin to engage in severe forms of NSSI earlier. Unfortunately, because of the limitations of our data, we do not know if NSSI precedes other manifestations of BPD (including difficulty regulating anger, impulsivity, affective instability). This is an important research question linked to the possible identification of NSSI as an early marker of BPD diagnosis, including a clear-cut distinction with transient and age related dyscontrolled behaviors.

Greater frequency and diversification of self-harm were associated with female gender, which greatly exceeded the proportion of males in this sample. Prior works have failed to identify significant gender differences in the frequency and diversification of NSSI in community samples [2], while female gender was associated with a greater likelihood of engaging in NSSI in a psychiatrically impaired outpatient sample [23]. More research controlling for mediating variables related to comorbid mental disorders is needed to verify whether gender actually represents a discriminant risk factor for NSSI.

As far as psychopathological features are concerned, this study seems to confirm the strong link between BPD diagnosis and NSSI in adolescent inpatients [22]. We also would like to stress a peculiarity of this study, consisting of a clinically significant representation of other Cluster B Personality Disorders (namely, Histrionic and Narcissistic) and Passive-aggressive personality disorder. These disorders share "dramatic", anxious and dysphoric traits with BPD. The emotionally dysregulated features of this sample of inpatient adolescents could account for the need to resort to NSSI for self-regulation [33-35]. A systematic examination of Axis II diagnoses among adolescents with a recent and early history of NSSI would be informative to clinicians working with psychiatric inpatients.

A second peculiarity of this sample concerns Axis I diagnoses. In other studies concerning adolescent inpatients and outpatients populations [33] a significant over-representation of MDD and PTSD was reported. It is noteworthy that none of the individuals in the sample met criteria for MDD or any Mood Disorder. However, more than half of the subjects reported clinically significant scores at the CDI. This finding could be related with the high prevalence and severity of externalizing conducts as a strong factor associated with admission. It may also suggest a potentially distinct subgroup of adolescent self-injurers (characterized by "sub-threshold" depression and severe emotional and behavioral dysregulation). Finally, as additional explanations we should consider the lower prevalence rates of mood disorders in Italian adolescents in comparison to other countries [36] and the attitude to underestimate "depression" as admission diagnosis in Italy [37]. Overall prevalence of mood disorders in our inpatients population in the period of the study was as low as 9%. The relationship between depression symptoms with NSSI is anyway confirmed by the significant associations between CDI scores and frequency and number of types of NSSI, a trend also showed by Jacobson and colleagues in their study [23] including only outpatients.

As far as the distinction between NSSI-SA and NSSI-only subjects is concerned, several considerations should be made.

**Figure 1:**
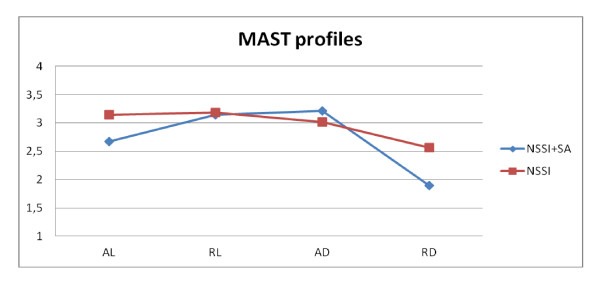
**The Figure shows the average scores of the 4 MAST subscales (AL: Attraction to Life, RL: Repulsion by Life, AD: Attraction to Death and RD: Repulsion by Death) in our sample: 1)NSSI-SA, 2)NSSI-only**.

Contrary to what could be expected neither depressive symptoms nor attitudes toward life and death significantly discriminate between NSSI-SA and NSSI-only subjects. In our opinion, however, these results are not enough to rule out the impact of such dimensions to understand the relationship between NSSI and suicidality. Three points should be considered in this regard. First, NSSI-SA subjects reported higher mean scores at both CDI and the MAST-AD and RL subscales (Figure 1). NSSI-SA patients also reported lower mean scores at the MAST-RD and AL subscales (Figure 1). Therefore, both CDI and MAST scores present a trend that distinguishes NSSI-SA from NSSI-only subjects in the expected direction. The absence of statistical significance could be due to the relatively small dimension of the sample, reducing the statistical power of the analysis.

Second, the AL mean scores present an inverse relationship with frequency and diversification of conducts. We would like to stress that these two indexes of NSSI are often reported [22-33] as key factors for predicting the escalation from NSSI to attempted suicide. It should be noted, that while depression and "Attitude toward life and death" definitely present a mutual statistical relationship, their impact on NSSI is direct and independent.

**Figure 2:**
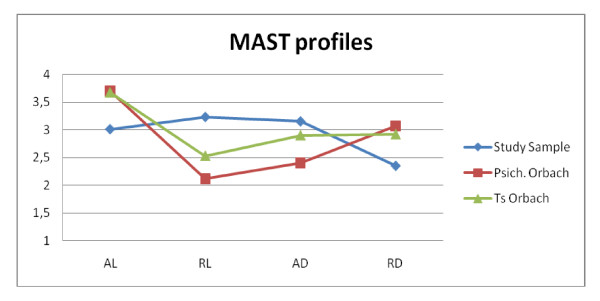
**The Figure shows the average scores of the 4 MAST subscales (AL: Attraction to Life, RL: Repulsion by Life, AD: Attraction to Death and RD: Repulsion by Death) in 3 samples: 1) NSSI patients (this study); 2) Suicidal Patients and 3) Psychiatric Non-suicidal Patients (Orbach's Study)**.

Third, it is important to highlight that if we compare the MAST scores of the overall sample of this study with the MAST scores obtained by suicide attempters from Orbach's study [28] our subjects should be considered at high risk for suicidal behaviors. In Figure 2, the MAST mean scores of our subjects are confronted with the mean scores from control and suicidal subjects from Orbach's original study. Given that suicide attempters from Orbach's study were significantly differentiated from normal controls and psychiatric non-suicidal patients by the MAST subscales (the statistical significance was for AL, RL, AD), the statistical differences would be even more accentuated for our subjects.

On the whole, some observations concerning the role of depressive symptoms and "Attitude toward life and death" should be made. In the absence of longitudinal data, no conclusions can be definitively drawn regarding to the role of these two variables to explain the escalation from NSSI to suicide attempts. However, the suicidal dimension of "Attitude toward life and death" as well as depressive symptoms are both related to higher frequency and diversification of NSSI. We might hypothesize that "Attitude toward life and death" may be the subjective outcome of the gradual habituation to pain and fear engendered by the high recurrence of NSSI, possibly facilitating suicidal behaviors as put forward by Nock's [22-34] model. Regardless of these important research questions, we would like to draw the attention on the clinical meaning of these results.

Notwithstanding the absence of statistical significant evidence, the relation between MAST and depressive symptoms with possible predictors of suicide attempt should lead clinicians to consider these two variables for a careful screening of NSSI populations with severe psychiatric impairment.

## Conclusions

To our knowledge, this is the first study including a sample of inpatient adolescents to analyze the role of the construct of "Attitude toward life and death" alongside psychopathological dimensions usually considered as risk factors for NSSI. Data from this study indicate that the understanding of NSSI and its relationship with suicidal behaviors in this population requires a complex evaluation. Previous research gives a somewhat inconsistent picture of the impact of NSSI in adolescence on suicidal behavior.

Limitations of this study should be addressed. A first limitation concerns the nature and the relatively small size of the sample as well as its composition. The current study examined NSSI and psychopathology in hospitalized adolescents and findings cannot be generalized to community samples. Severity of psychopathology and prevalence of personality disorders observed may be higher than in outpatients samples or other inpatients populations. Nevertheless, these data provide useful informations for those working in acute psychiatric settings and a point of departure for future research: identification of high-risk groups and subsequent early intervention strategy is one of the most demanding tasks in adolescent psychiatry. Second, our data relied on cross-sectional observations and retrospective self-report data. Hypothesis on causal relation between the observed psychopathological features and the actual risk for suicide requires prospective research and represents a direction for future work. Our research proposes one possible developmental association leading from early NSSI to suicide attempts; this hypothetical pathway should be confirmed by further empirical evidence based on follow-up studies.

Professionals involved in treating adolescent inpatients should take into account treatment options more addressed to manage BPD features in NSSI adolescents more than depressive symptoms. Many adolescents who self-injure do not search treatment, and the possibility to evaluate risk may be limited to emergency or intensive care units. Information from these settings may be useful for all clinicians working in adolescent mental health services given the well known clinical *continuum *in adolescence between diffuse behaviors and severe psychopathology. Findings from this study should prompt clinicians to evaluate not only internalizing disorders in association with NSSI, but the combination of emotional dysregulation and reduced "attraction to life" attitudes.

## Competing interests

The authors declare that they have no competing interests.

## Authors' contributions

All authors contributed equally to this work. All authors read and approved the final manuscript.
